# High field superconducting properties of Ba(Fe_1−*x*_Co_*x*_)_2_As_2_ thin films

**DOI:** 10.1038/srep17363

**Published:** 2015-11-27

**Authors:** Jens Hänisch, Kazumasa Iida, Fritz Kurth, Elke Reich, Chiara Tarantini, Jan Jaroszynski, Tobias Förster, Günther Fuchs, Ruben Hühne, Vadim Grinenko, Ludwig Schultz, Bernhard Holzapfel

**Affiliations:** 1 IFW Dresden, P.O. Box 270116, 01171 Dresden, Germany; 2 Karlsruhe Institute of Technology, Institute for Technical Physics, 76344 Eggenstein-Leopoldshafen, Germany; 3 Nagoya University, Department of Crystalline Materials Science, Graduate School of Engineering, Nagoya 464-8603, Japan; 4 Dresden University of Technology, Faculty for Natural Science and Mathematics, 01062 Dresden, Germany; 5 NHMFL, Florida State University, Tallahassee, Florida 32310, USA; 6 HZDR, Dresden High Magnetic Field Laboratory, 01328 Dresden, Germany

## Abstract

In general, the critical current density, *J*_c_, of type II superconductors and its anisotropy with respect to magnetic field orientation is determined by intrinsic and extrinsic properties. The Fe-based superconductors of the ‘122’ family with their moderate electronic anisotropies and high yet accessible critical fields (*H*_c2_ and *H*_irr_) are a good model system to study this interplay. In this paper, we explore the vortex matter of optimally Co-doped BaFe_2_As_2_ thin films with extended planar and *c*-axis correlated defects. The temperature and angular dependence of the upper critical field is well explained by a two-band model in the clean limit. The dirty band scenario, however, cannot be ruled out completely. Above the irreversibility field, the flux motion is thermally activated, where the activation energy *U*_0_ is going to zero at the extrapolated zero-kelvin *H*_irr_ value. The anisotropy of the critical current density *J*_c_ is both influenced by the *H*_c2_ anisotropy (and therefore by multi-band effects) as well as the extended planar and columnar defects present in the sample.

The intermetallic Fe-based superconductors (FBS) of ThCr_2_Si_2_-type crystal structure, such as electron or hole-doped *AE*Fe_2_As_2_ (*AE* alkali earth metal), are characterized by a low Ginzburg number 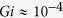
1,2, high upper critical fields, 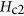
3, and very low 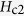 anisotropies, 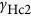
4,5, which tend to approach 1 at low temperatures. Although the in-plane coherence length, 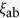, is similarly small as in YBa_2_Cu_3_O_7−δ_ (YBCO), grain boundaries (GBs) are slightly less detrimental to the current flow than in high 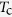 cuprates^6^. The reason is mainly a difference in order parameter symmetry, being predominantly *d*-wave in YBCO but of *s*-wave type (presumably with a sign change, *s*±)^7^ in FBS. All these properties make FBS materials interesting for high-field applications at low temperatures since they combine advantageous properties of the low-temperature superconductors (narrow in-field transition, low *Gi*, low 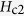 anisotropy) and high- 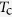 cuprates (high upper critical fields 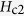). Indeed, first prototypes of Co-doped BaFe_2_As_2_ (Ba-122) coated conductors^8^,^9^ and K-doped Ba-122 powder-in-tube wires^10^,^11^ have been reported.

A well-investigated member of this family is Co-doped Ba-122 due to its relatively easy preparation as thin films by pulsed laser deposition and stability at ambient conditions^12^. Furthermore, Co-doped Ba-122 proved to be very susceptible to a high density of artificial pinning centers, and record values of the maximum pinning force density, 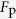, of around 70 GN/m^3^ for 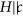 at 4.2 K have been reported for films on CaF_2_ substrate^13^. Lee *et al.* demonstrated that Co-doped Ba-122 films grown at relatively high O_2_ partial pressure^14^ or with oxygen-containing targets^15^ develop correlated *c*-axis-oriented columnar oxide defects. These defects lead to a huge contribution to pinning for fields applied parallel to the *c*-axis^16^. Katase *et al.* found similarly high *J*_*c*_ values for Co-doped Ba-122 on (La,Al)(Sr,Ta)O_3_ (LSAT)^17^. In most of these pinning-improved Co- or P-doped^18^,^19^ Ba-122 samples, a large density of *c*-axis correlated or extended random defects is introduced. This paper investigates the high-field transport properties of Co-doped Ba-122 thin films with several different natural growth defects, such as small-angle grain boundaries and stacking faults. It will be shown how these defect populations, in combination with the multi-band superconductivity, influence the vortex matter and the pinning properties in different regions of the *H*-*T* phase diagram.

## Results and Discussion

### Microstructure

**Figure 1:**
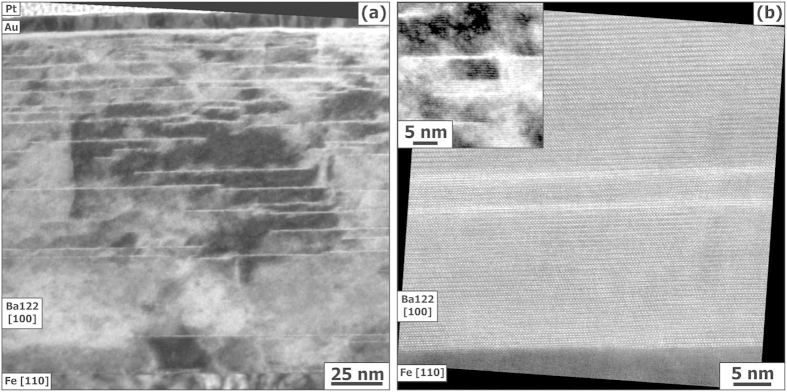
(**a**) Bright field TEM image of the lamella showing a large number of stacking faults parallel to the *ab*-plane. (**b**) High-resolution TEM image of two stacking faults, i.e. missing FeAs layers. The inset (bright field) shows the diffraction contrast image of a tilted grain (in-plane) between two stacking faults which strongly suggests the existence of *c*-axis correlated defects.

The film investigated grew phase-pure and highly textured with in-plane and out-of-plane full width at half maximum, FWHM, of 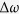 = 0.74° and 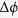 = 0.9°, Suppl. S1. The sample, however, does contain a large density of *ab*-planar defects, as revealed by transition electron microscope (TEM) images of focused ion beam (FIB) cuts near the microbridges, Fig. 1. These defects are presumably stacking faults (i.e. missing FeAs layers)^20^. The reason for this defect formation (also observed on technical substrates)^21^ is not fully understood. Possible reasons are a partial As loss during deposition^22^, and relaxation processes in combination with the Fe buffer layer^23^. Estimating the distance between these intergrowths leads to values varying between 5 and 10 nm. Between the planar defects, an orientation contrast is visible in TEM (inset Fig. 1b), i.e. the brighter crystallites are slightly rotated either around (010) (out-of-plane spread, 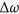) or around (001) (in-plane spread, 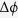) and enclosed by dislocation networks or small-angle GBs. Since the crystallites are sandwiched between planar defects, an in-plane misorientation is most likely. The out-of-plane misorientation, on the other hand, is visible as a slight tilt of the *ab*-planar defects with respect to each other, especially in the upper part of the sample. No globular or columnar precipitates were found.

### Upper critical field *H*_c2_

**Figure 2:**
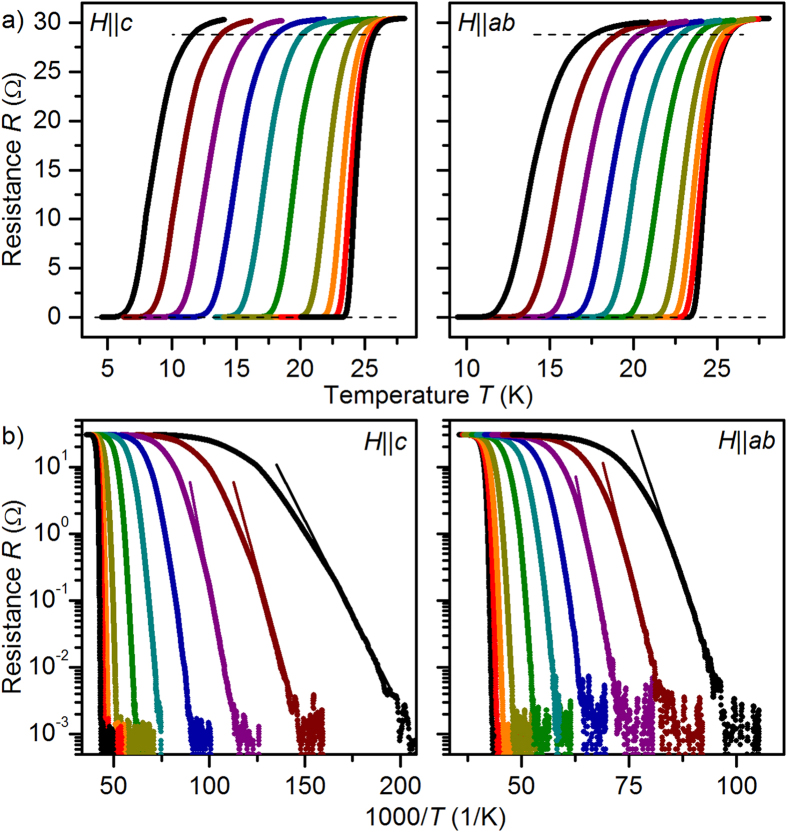
Resistive transitions *R*(*T*) measured in static magnetic fields up to 35 T for both major directions 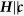 (left) and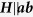 (right). (**a**) linear representation. The broken lines indicate criteria for determining the upper critical field (95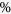) and irreversibility field (1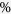), respectively. (**b**) Arrhenius plots.

**Figure 3:**
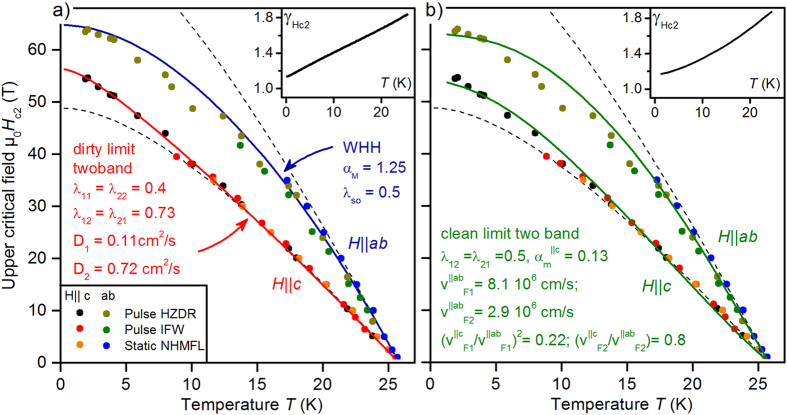
Temperature dependence of the upper critical field *H*_c2_. It was determined with a criterion of 95% *R*_n_ of resistance curves in pulsed (HZDR and IFW) and static magnetic fields (NHMFL). The dashed lines are fits near 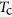 with single-band orbital limitation. 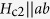 shows clear indication of paramagnetic limitation, 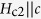 of two-band behavior, which can reasonably be fitted both in the dirty (**a**) and the clean limit (**b**). Insets: Temperature dependence of the respective 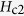 anisotropies 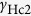.

To determine the nature of the superconducting transition, the temperature dependent resistance 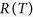 has been measured in static magnetic fields up to 35 T for both major directions, 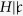 and 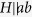, Fig. 2a, as well as 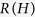 in pulsed fields up to 65 T (Suppl. S2). 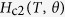 was estimated from the 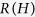 experimental data using a constant 95% 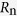 criterion (upper dashed line in Fig. 2a). A 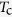 of 25.8 K in absence of an applied magnetic field was recorded. The temperature dependence of 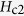, Fig. 3, shows different behavior for 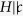 and 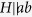. Both directions differ clearly from the single-band WHH model for orbital limitation (dashed lines). For 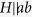, 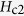 can be fitted well by the single-band WHH model including paramagnetic limitation and spin-orbital effects. Best fits were achieved with a Maki parameter 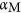 = 1.25 and a spin-orbit scattering term 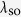 = 0.5. Conversely, for 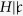 a two-band approximation is necessary since the orbital 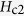 is lower than the measured 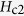 values. The data were analyzed using the WHH approach for two-band superconductors in the dirty^24^ and the clean limit^25^,^26^. In the dirty limit, a reasonable description of the experimental data was achieved with the diffusivity, scattering parameters and coupling constants given in Fig. 3a. Similar fits have been shown for single-crystal data^5^.

**Figure 4:**
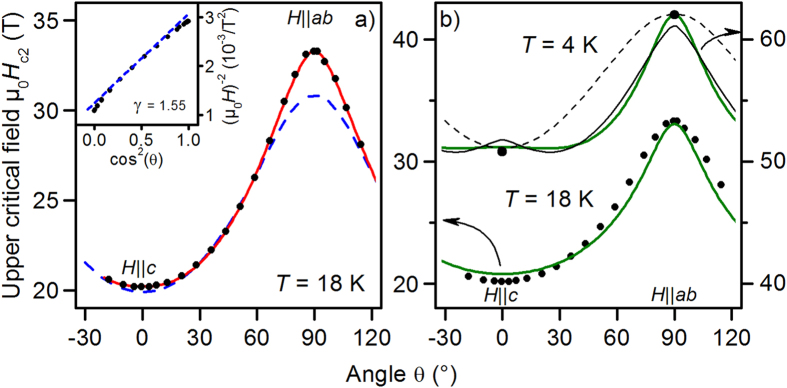
Angular dependence of the upper critical field *H*_c2_ at 18 K and 4 K. (**a**) The blue dashed line shows the result of a linearization of the one-band AGL formula (Eq. 2, 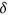
=2), inset. Deviations near 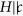 and 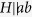 are clearly visible. Red line: phenomenological approximation of 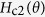 with Eqs 1,2 (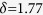). (**b**) Solid green lines: calculations using the clean-limit two-band WHH model with the same parameters as shown in Fig. 3. For 4 K, a fit according to Eqs 1,2 (full black line, → 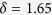) and a one-band AGL dependency (dashed) are also shown.

However, as pointed out by Gurevich, most of iron-pnictide superconductors should be in the clean limit (electron mean free path 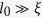) due to low Fermi velocities^25^,^26^. The observed scaling behavior of the slope of 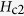 at 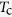, 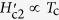, for many iron pnictides supports this assumption^27^. Applying the two-band description in the clean limit including paramagnetic effects leads to reasonable fits for the temperature dependencies of 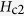 for both crystallographic directions, Fig. 3b, and angular dependencies 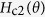 at constant temperature, Fig. 4b. For the calculations, we used moderate values of the coupling constants (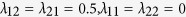) consistent with the case of 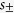-wave superconductivity in this compound. Also, we used values of the Fermi velocities in agreement with average values obtained from ARPES data for the BaFe_2_As_2_ system^28^. Paramagnetic effects for both directions were taken into account by introducing small values of the Maki parameters. Here, we assumed that the anisotropy of the Maki parameters is given not only by the an-isotropy of the Fermi velocities but also by the anisotropy of the spin susceptibilities. This was taken into account by introducing the anisotropy of the magnetic moments in plane and out of plane. Reasonable agreement of the calculations with experimental data were obtained for 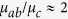.

The clean- and dirty-limit 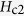 anisotropies, 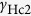, insets Fig. 3, are increasing almost linearly from 1.2 near 0 K to 1.85 close to 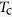. This is a combined effect of multi-band superconductivity and paramagnetic limitation. The sample investigated here shows at all temperatures a slightly lower 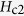 anisotropy than microstructurally clean films of similar nominal composition^29^. Whether this effect is a direct consequence of the defect structure or caused by a slightly different doping state due to e.g. As disorder or differing Co content could not been clarified completely.

The angular dependence of the upper critical field, 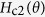, shown in Fig. 4a at 18 K, cannot be described in the full angular range by the Anisotropic Ginzburg Landau (AGL) formalism for one-band 3D superconductors. This is illustrated by a linearization method of the AGL dependency as proposed by Tarantini *et al.*^30^ [inset Fig. 4a]. The resultant anisotropy curve at 18 K, shown as blue dashed line in Fig. 4a, deviates from the measured 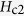 values for field directions close to 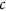 and 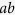. The deviation for 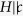 seems to be marginal in 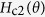. It is, however, not negligible for a correct determination of the (AGL) anisotropy parameter, as evidenced by the linearization method.

For (single-band) dirty limit, Takezawa *et al.* showed that periodic structures of superconducting and non-superconducting layers can lead to intermediate functions between 3D AGL and pure 2D behavior^31^. This effect had also been reported by Ghosh *et al.* for CaAlSi single crystals with planar defects similar to the ones seen in our sample^32^. This can be explained by a modification of the coherence length via a reduction of the mean free path 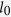
33. Both kinds of defects in our sample, GB network and 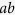-planar intergrowths, would lead to different angular dependencies of 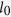 resulting in a modified 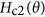 dependency. It is feasible that in dirty limit both effects, multi-band superconductivity and extended defects, together determine the exact 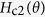 dependence.

Alternatively, the 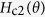 dependencies can be explained solely by a combination of multi-band and paramagnetic effects, assuming that the film is in the clean limit. In Fig. 4b, the result of the two-band calculation with the same parameters as given in Fig. 3b are shown for 18 K and 4 K. For 18 K, the deviation between fit and data especially around 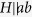 compared to the AGL fit is decreased considerably. For 4 K, a small secondary peak around 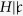 for the clean-limit 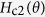 is observed, which is a consequence of the anisotropic paramagnetic effects. Which scenario, clean or dirty limit, holds for this sample is not possible to determine at the present stage since both yield fits of 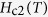 with similar accuracy and the 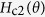 dependencies are expected to be similar as well. Future experiments regarding coherence length and mean free path in samples with and without extended defects should clarify this issue.

For further investigations of the 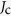 anisotropy, see below, it is helpful to describe 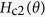 with some well-behaved analytical function. One possibility is:


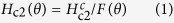



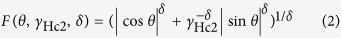


where 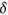 is a free parameter close to 2 and 
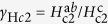
. With *δ* →2, Eq. 1 goes back to the usual AGL dependency. The full red line in Fig. 4a is a fit to this function with *δ* = 1.77 and 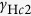 = 1.65. Also the clean-limit 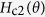 dependence at 4 K can be approximated reasonably with this function, Fig. 4b full black line.

### Thermal activation

Arrhenius plots, ln *R* (1/*T*), Fig. 2b, show the typical linear behavior of thermal activation of flux motion. The activation energy 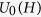 for vortex motion can be estimated under the assumption of linear temperature dependence, 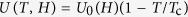, which leads to ln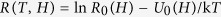 and ln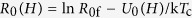, where 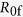 is a prefactor. The 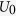 data were refined by evaluating 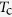 and 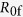 from linear fits of ln 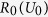 and by fixing the crossing point 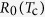 of the linear fits in the Arrhenius graphs, Fig. 2b. This method yields a 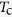 value of 25.8 K, corresponding to 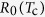 = 95% 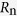 (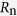 normal state resistance) in accord with the criterion used for evaluating 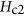.

**Figure 5:**
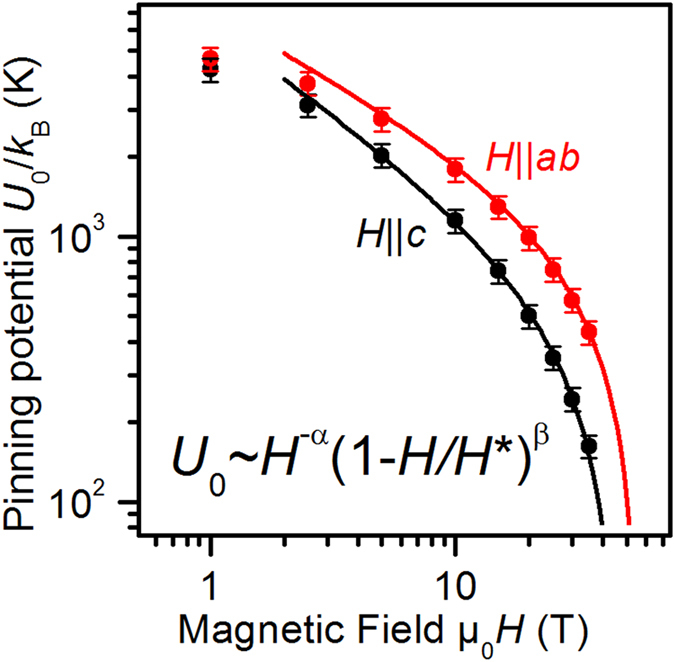
Magnetic field dependence of the pinning potential *U*_*0*_. Full lines are fits to Eq. 3 for *H* ≥ 5 T.

The field dependencies of 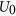 for both major directions, Fig. 5, are best described by


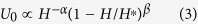


This functionality was first proposed for the case of MgB_2_ by Thompson *et al.*^34^ who argued that the exponents should be similar to the exponents in the field dependence of the pinning force density. As will be shown later, this is, interestingly, not the case for 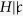 in our sample. Eq. 3 can further be explained by statistical considerations. A statistical distribution of pinning energies in the sample and the principle of entropy maximization also lead naturally to this dependency. In that case, it takes the form of a beta-distribution, i.e. the maximum-entropy distribution over a finite range (here magnetic field range)^35^ because 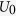 has to go to zero near 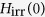, i.e. 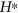. The data are well fitted above 5 T with exponents*α* = 0.72 and 0.5 for 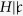 and 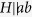, respectively, and *β* = 1.0 for both field directions. 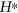 was varied independently, best fits were achieved with (46
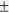
1) T and (56±0.4) T for 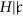 and 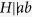, respectively. At low fields, the data differ from fits to Eq. 3 because of the crossover to the regime of single vortex pinning where 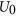 is constant. Similar 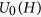 dependencies have recently been found for polycrystalline MgB_2_, NbSe_2_^36^, and FeSe^37^,^38^, as well as LiFeAs single crystals^39^.

### Critical Current Density *J*_c_

**Figure 6:**
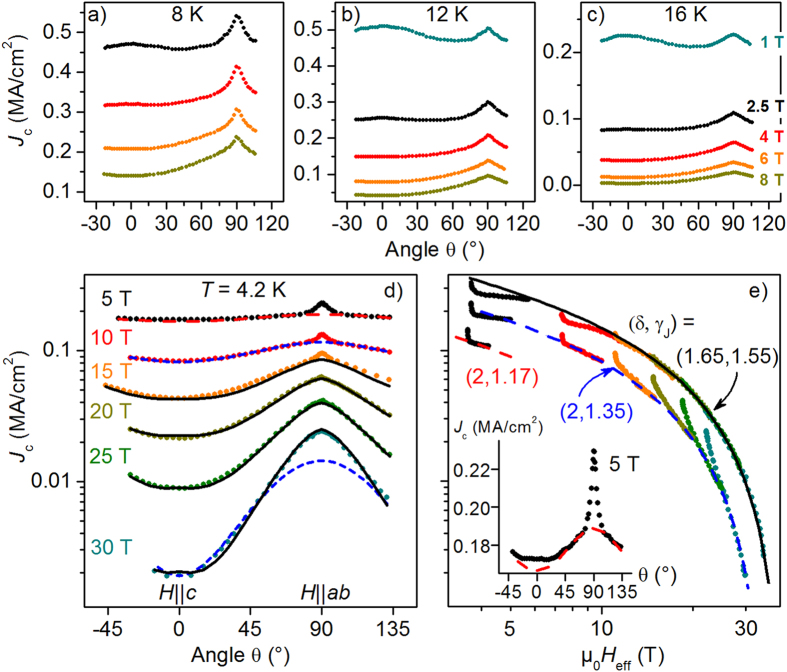
Angular dependence of *J*_c_. (**a**) 8 K, (**b**) 12 K, (**c**) 16 K showing a 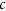-axis peak at low magnetic fields besides the always present 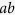 peak. (**d**) 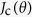 at 4.2 K for several fields up to 30 T in logarithmic representation. The full and broken lines in (**d**,**e**) represent the “random-pinning contribution”, Eq. 6, and are the same for all three types of scaling. (**e**) 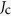 scaling applying scaling function 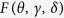 (Eq. 2, full black lines) as well as scaling function 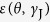 (Eq. 5, identical to 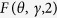, broken red and blue lines). The upper (black line) and lower data sets (red line) are shifted in 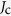 and 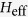 for clarity. Inset: 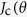, 4.2 K, 5 T) in linear representation.

The angular dependencies of the critical current density 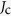 at low fields reveal a strong influence of the *c*-axis correlated defect structures, Fig. 6a–c. All 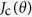 curves show a maximum at 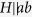 (*θ* = 90°). Additionally, there is a broad maximum visible at 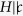 (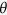 = 0°) for the respective lowest fields. The 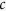-axis peak vanishes at all temperatures around 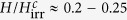 (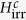 being the irreversibility field 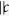 determined from 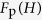 fits) and grows with decreasing 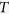 at a given magnetic field. The curve for 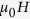 = 1 T at 12 K represents the lowest 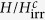 value of 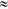
0.06 in the data set of Fig. 6a–c and shows the highest 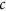-axis peak [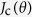 at 1 T, 8 K was not measureable due to current limitation]. A similarly broad 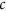-axis peak related to threading dislocations has recently been found in P-doped Ba122 thin films by Sato *et al.*^19^ This increasing *c*-axis pinning for lower *T* is in contrast to the behavior in cuprates such as YBCO where the *c*-axis peak has been observed to disappear at low *T*^40^. This striking difference has two reasons: Firstly, the mass anisotropy in YBCO is much larger and so the *ab*-peak is growing disproportionately with decreasing temperature, hiding a minor *c*-axis peak. Secondly, Ba-122 is a multi-band superconductor in contrast to YBCO. Therefore, the anisotropy of the penetration depth, 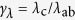, does not equal the 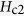 anisotropy, 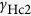. With 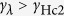, as for Ba-122, any strong pinning contribution at large random defects will lead to a maximum in the pinning potential for 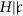
41. The larger the distance to the irreversibility line (i.e. the lower the flux creep processes), the stronger this contribution is and the better it can compete with the *ab* contribution.

**Figure 7:**
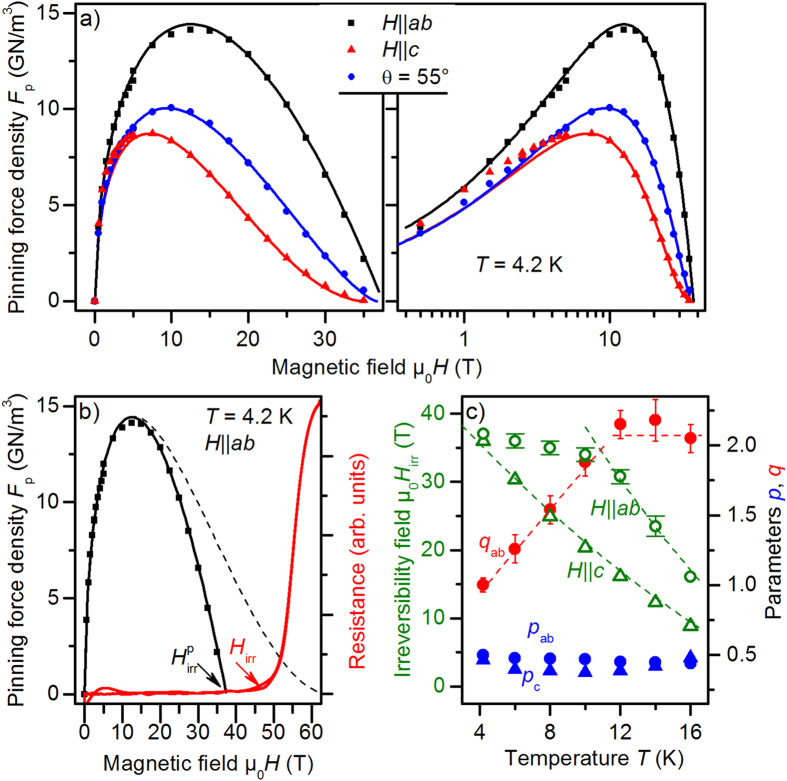
(**a**) Pinning force density 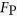 at 4.2 K for three magnetic field directions in linear and logarithmic field scale. The lines are functions according to Eq. 7 with parameter sets 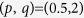 for (**c**), (0.5,1.45) for 55°, and (0.5,1) for 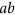. Below 5 T, (**a**) 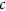-axis contribution is visible. (**b**) Magnetic field dependence of the pinning force density 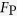 and of the resistance 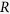 illustrating the region of very weak pinning at high magnetic fields. The dashed line is a low-field fit with 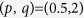 similar to high 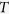 as well 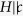. (**c**) Temperature dependence of 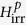 and the parameters 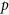 and 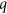: comparison between 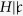 and 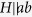, 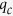 was fixed at 2.

**Figure 8:**
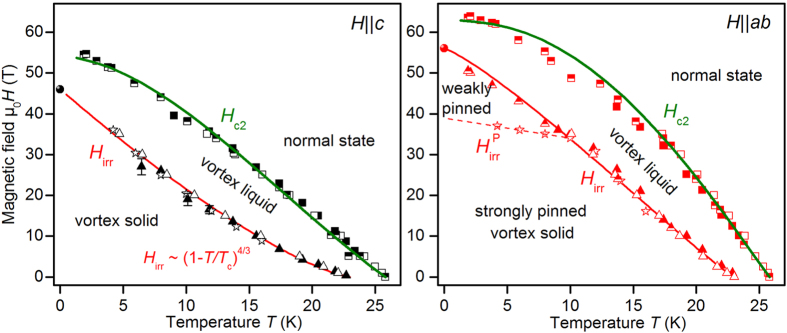
*H*-*T* phase diagram of a Co-doped Ba-122 thin film with extended defects for 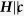 and 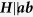. Squares: upper critical field 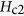, triangles: resistive irreversibility field 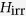, stars: pinning-force irreversibility field 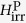, balls: 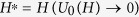 (*cf.* Fig. 5a). Different symbol fillings indicate different measurements (pulsed (open and half symbols) or static fields (full symbols)), green lines: clean-limit two-band fits.

As shown in refs 42 and 43 *J*_*c*_ of microstructurally clean samples (i.e. containing very few defects of size larger than the coherence length) of multi-band superconductors is scalable on an effective magnetic field


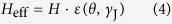



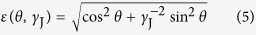


with a single, yet temperature-dependent scaling parameter 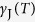, which was identified as 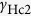. This approach follows the Blatter-Geshkenbein-Larkin (BGL) scaling for anisotropic single-band superconductors and can be understood as transformation of the anisotropic superconductor into an equivalent isotropic one^44^. Commonly, the envelope function of the scaled *J*_*c*_ data is associated with pinning at isotropic and random defects^45^. Fig. 6d displays the angular dependence of *J*_*c*_ at 4.2 K for several magnetic field strengths between 5 and 30 T. The same data are scaled according to Eq. 4 in Fig. 6e (lower data sets, dashed lines). The envelope, and simultaneously the corresponding random-pinning contributions in Fig. 6d, was fitted using an empirical function^46^ which can be understood as combination of the modified Kim model^47^ and the generalized Kramer model^48^.


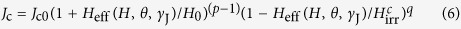


by independently varying the parameters 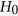, 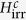, and 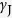. The parameters 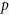 and 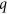 were fixed at 0.5 and 2, respectively. These values correspond to the fit parameters 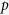, 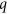 of the pinning force density discussed below, Fig. 7 and Eq. 7, and are well justified for pinning at small random defects^43^. 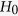 is related to the self-field^49^ and/or the accommodation field for single vortex pinning^50^. Good fits were achieved with parameters 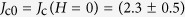 MA/cm^2^ (slightly larger than the measured value), 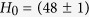 mT, 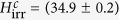 T (in good agreement with the value determined by resistive measurements at 4.2 K, Fig. 8), and 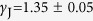, which is slightly larger than 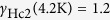.

The data above 10 T can be well scaled in the vicinity of the 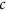-direction using this formalism. However, there is a large intermediate angular range which neither follows the scaling nor really belongs to the sharp 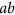-peak due to correlated 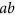-planar defects. This region can be scaled if one takes again into account the altered angular dependency of 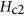. If, instead of 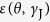, Eq. 5, 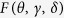, Eq. 2, with 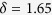 found for 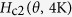 (clean-limit two-band approximation, Fig. 4b) is used for the scaling, nearly the full angular range of 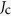 can be scaled for high fields, as illustrated in Fig. 6e upper data sets (full line, data shifted for clarity) and Fig. 6d (full lines). One has to keep in mind, however, that both parameters 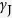 (which still differs from 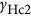) and 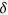 are purely phenomenological here. The scaling merely shows that *J*_*c*_ at high fields is determined by the angular dependence of 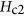 since in this region correlated defects play a minor role. The sharp extra peak near *ab* at fields below 20 T is mainly due to correlated pinning at the planar defects visible in TEM and, therefore, not following either scaling.

Whereas for high fields the modified scaling according to 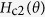 gives the better scaling results, the usual BGL scaling is better at low fields, as illustrated in Fig. 6d for 10 T. In fact, for 5 T, 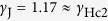 results in near perfect scaling behavior for intermediate angles, Fig. 6d,e (red dashed line and inset). For 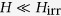, the angular dependence of 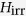 plays a minor role and the contributions of random and correlated pinning can, with necessary care, be distinguished in the common way. The difference between measured *J*_*c*_ and scaling curve at 5 T near 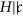 is again due to the dislocation networks and disappears between 5 and 10 T, i.e. at 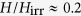, *cf.* the disappearance of the *c*-axis peak in Fig. 6a–c.

### Pinning Force Density *F*_p_ and Phase Diagram

The field dependence of the pinning force density 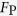 depends strongly on temperature and field orientation, Fig. 7. All data for 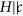 and 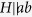 between 4.2 and 16 K as well as for 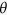 = 55° at 4.2 K were fitted with the empirical formula^51^:


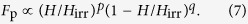


For 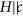, 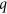 was fixed at 2.0 to minimize fit ambiguity, and 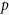 was slightly lower than 0.5 for all temperatures due to the correlated 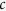-axis defects active at low fields. In Fig. 7a, the difference between the fit function with parameter set 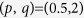 and the 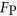 values illustrates this influence at low fields, as emphasized in logarithmic scale. In contrast to 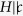, the parameter 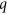 is lower than 2 for 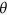 = 55° (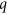 = 1.45) and 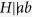 (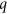 = 1.0) at 4.2 K. Below 5 T, 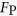 for 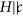 exceeds the 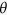 = 55° data, again a consequence of the 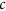-axis correlated defects.

As summarized in Fig. 7c, 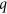 is around 2 at high temperatures and decreases roughly linearly to 1 below 12 K for 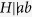. This matches the temperature where the irreversibility field 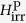, determined from the 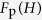 dependence, shows a sharp kink. Below 12 K, 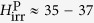 T is distinct from the 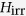 values determined resistively for 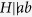, Fig. 8. A parameter set 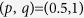 as determined for 4.2 K is typical for core pinning at extended planar defects^52^. A matching field of 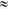
35-37 T corresponds to a defect distance of around 7.5 nm, which is in good agreement with the distance of the 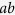-planar defects estimated by TEM (5–10 nm), Fig. 1. Vortices at higher magnetic flux densities are only pinned very weakly. A fit with 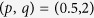 leads to unreasonably high 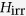 values (dashed line Fig. 7b). In comparison, a clear change in pinning mechanism at low temperatures is hard to observe in cuprates due to the high irreversibility fields. Nevertheless, it can be assumed that these effects may be present in cuprates with similar microstructures.

The magnetic field-temperature phase diagram, Fig. 8, shows clear differences for 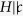 (left) and 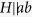 (right): For 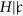, the irreversibility field 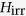 can be well described by a simple power law of the form 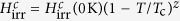 with 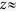 4/3 (red line). Similar power laws for 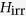 at high temperatures have been measured for Co-doped Ba-122 single crystals by resistive^53^ and AC susceptibility measurements^54^. In contrast, 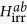 seems to be strongly influenced by the temperature dependence of 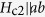 as well as the extended planar defects. It was fitted empirically as 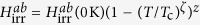 with 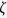 = 1.2 and 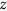 = 1.08. For both directions, 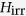 is distinct from the upper critical field at zero temperature, 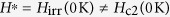. That means the sample investigated shows a clear and relatively wide vortex-liquid region at zero temperature despite the relatively low Ginzburg number of Ba-122 compounds (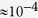). The origin of this broad region of the vortex-liquid phase is unclear and has to be clarified in further investigations.

## Conclusion

The electrical transport properties of a superconducting Co-doped Ba-122 thin film with a large density of 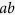-planar defects (stacking faults or intergrowths) were measured in high magnetic fields. Between these planar defects, a dense network of small-angle grain boundaries parallel to 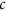 is formed. Both types of defects contribute to strong pinning in such films, indicated by the presence of a clear 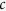-axis peak in 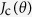 in fields up to around 5 T and a sharp 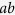 peak above the random-pinning curves up to around 20 T. The angular dependence of 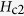 was found to be influenced by the multi-band superconductivity and/or the extended defects. This, in consequence, strongly influences the angular dependence of the critical current density,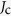. 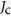 can be scaled very effectively by a modified Anisotropic Ginzburg-Landau approach which takes into account the actual dependence of 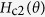. The vortex-matter phase diagram down to lowest temperatures was evaluated for both major directions, 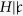 and 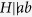, from resistive measurements in magnetic fields up to 62 T. 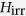 was measured in the complete superconducting region, revealing a surprisingly large vortex-liquid phase at zero temperature. 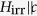 follows a simple power law whereas 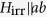 is influenced by the temperature dependence of 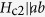 as well as the presence of planar defects. At fields above the matching field of the planar defects (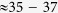 T), the vortices are only weakly pinned at random disorder. All data were analyzed self-consistently: Arrhenius derivation of the pinning potential 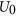 and determination of 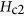 used the same 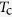 value. Furthermore, 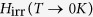 corresponds to 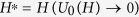.

## Methods

### Thin film preparation

Co-doped Ba-122 thin films of 170 nm thickness were grown by pulsed laser deposition under UHV conditions (base pressure 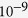 mbar) from a stoichiometric sintered target of Ba(Fe_0.92_Co_0.08_)_2_As_2_ using a KrF excimer laser (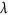 = 248 nm). The energy density of the laser beam at the target was around 4 J/cm^2^, the repetition rate 10 Hz, the substrate temperature 750 °C. As substrate, we used single-crystalline (001) MgO covered by an epitaxially grown 20 nm thick (001) Fe layer. The Fe layer was grown at room temperature and 5 Hz repetition rate and subsequently heated to the deposition temperature of Ba-122 (750 °C) to ensure a smooth surface^55^.

### Microstructural Analysis

Crystal structure, texture, and phase purity were measured by x-ray diffraction (XRD) in parallel-beam geometry with Co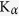 radiation (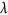 = 1.78897 Å) on a Bruker D8 Advance, and in Bragg-Brentano geometry on a Phillips X’pert goniometer with an Euler cradle using Cu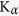 radiation (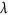 = 1.54056 Å). The microstructure was further investigated by transmission electron microscopy (TEM) on an FEI Tecnai T20 (LaB_6_, 200 kV) and an FEI Titan 80–300, operating at 300 kV with an image 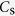 corrector. The lamella was prepared with the *in-situ* lift-out method in a focused ion beam (FIB) device^56^.

### Electrical transport properties

Resistance 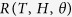 and critical current density 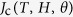 in dependence of temperature, *T*, magnetic field, *H*, and its orientation, *θ*, were measured on microbridges in four-point geometry in maximum Lorentz force configuration. The angle *θ* is measured between the magnetic field and the crystallographic 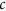-direction. The microbridges, fabricated by Ar ion etching and laser cutting, had widths between 50 and 250 μm and lengths between 0.5 and 1 mm. PPMS devices with fields up to 9 T and 16 T, a Florida NHMFL Bitter magnet up to 35 T, and the Dresden pulsed field facilities at IFW Dresden^57^ (45 T) and HZDR Rossendorf 58 (62 T) were used for these measurements. The upper critical fields 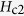 as well as the critical temperature 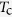 were determined at several constant levels around 90% of the normal-state resistance 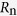 at 35 K of the Fe/Ba-122 bilayer and checked for plausibility regarding curvature of 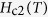 near 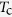 and regarding 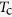 values determined from thermal activation of flux motion and scaling of 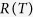 in the vicinity of a hypothetical glass-liquid transition (not discussed in this paper). A value of 95% 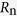 showed consistency. This is slightly higher than the usually applied 90% 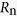 due to the conducting Fe interlayer, as described in ref. 59. The irreversibility field 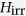 was estimated from the field dependence of the pinning force, 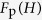 in combination with a plausibility check of the 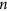-value (exponent in the electric field-current density characteristics 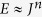 near 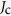: 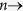 1 for 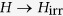). The critical current density was determined with a constant electrical field criterion of 1 μV/cm from 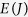 characteristics. The validity of the 95% 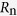 criterion for 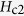 as well as the phenomenological fit function Eqs. 1 and 2 was further checked by rescaling 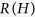 at several angles *θ* to 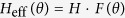, Suppl. S3.

## Additional Information

**How to cite this article**: Hänisch, J. *et al.* High field superconducting properties of Ba(Fe_1−*x*_Co_*x*_)_2_As_2_ thin films. *Sci. Rep.* **5**, 17363; doi: 10.1038/srep17363 (2015).

## Supplementary Material

Supplementary Information
