# Supplementary material for: High field superconducting properties of Ba(Fe1−xCox)2As2 thin films
Source: Sci Rep. 2015 Nov 27;5:17363. doi: 10.1038/srep17363 (PMC4661601; doi:10.1038/srep17363)
Supplement: Supplementary Information [file srep17363-s1.pdf]

Supplement – High field superconducting properties of $Ba(Fe_{1-x}Co_x)_2As_2$ thin films

Jens Hänisch[1,2,*], Kazumasa Iida[1,3], Fritz Kurth[1,4], Elke Reich[1], Chiara Tarantini[5], Jan Jaroszynski[5], Tobias Förster[6], Günther Fuchs[1], Ruben Hühne[1], Vadim Grinenko[1,3], Ludwig Schultz[1,4], and Bernhard Holzapfel[2]

[1]IFW Dresden, P.O. Box 270116, 01171 Dresden, Germany
[2]Karlsruhe Institute of Technology, Institute for Technical Physics, 76344 Eggenstein-Leopoldshafen, Germany
[3]Nagoya University, Department of Crystalline Materials Science, Graduate School of Engineering, Nagoya 464-8603, Japan
[4]Dresden University of Technology, Faculty for Natural Science and Mathematics, 01062 Dresden, Germany
[5]NHMFL, Florida State University, Tallahassee, Florida 32310, USA
[6]HZDR, Dresden High Magnetic Field Laboratory, 01328 Dresden, Germany
[*]jens.haenisch@kit.edu

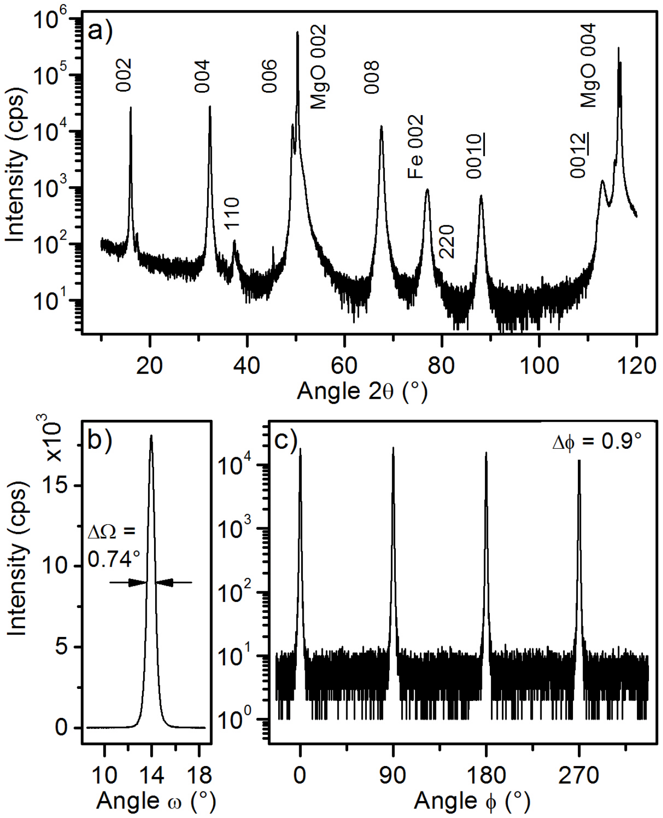

Figure S1. X-ray diffraction of the Ba-122/Fe/MgO sample: a) θ-2θ scan showing mainly c-axis orientation of Fe and Co-doped Ba-122 and a small component of the Co-doped Ba-122 (110) orientation, b) Co-doped Ba-122 (004) rocking curve, c) Co-doped Ba-122 (103) ϕ scan.

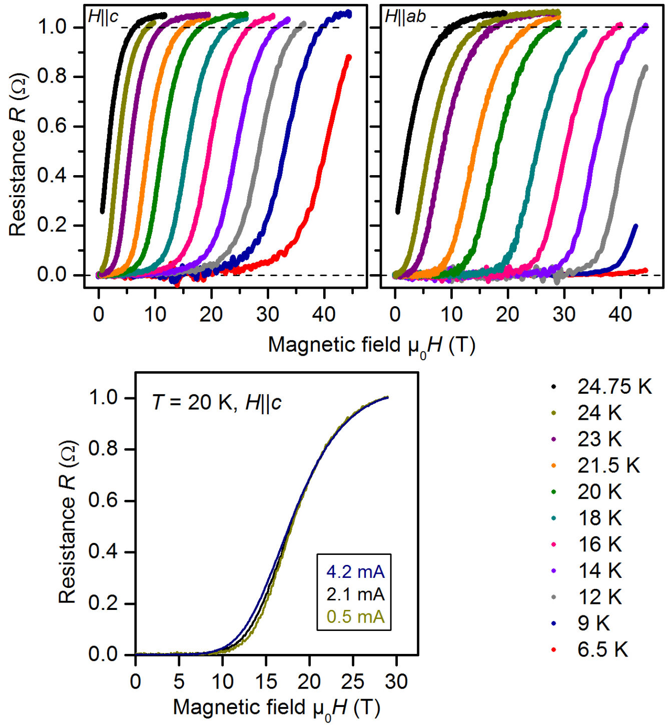

Figure S2. Field dependence of the resistance R for both major crystallographic directions, $H\|c$ and $H\|ab$, measured at the IFW Dresden pulsed field facility. The current test at 20 K, $H\|c$ (below) showed no heating effects. Differences below midpoint of transition are due to flux motion and correspond to the $J_c(H)$ dependence in the vicinity of H_{irr} (0.5 mA $\approx$ 300 A/cm^2).

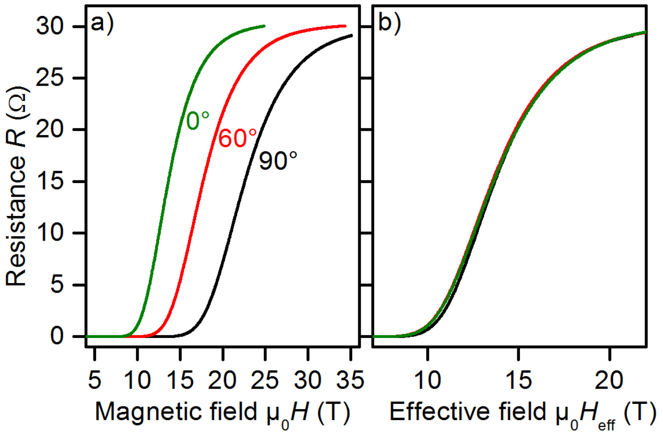

Figure S3. a) Magnetic field dependence of the resistive transition for three field orientations, b) Scaling of $R(H)$ according to $H_{eff}(\theta) = H \cdot F(\theta)$ (Eq. 2) with δ = 1.77. Above midpoint of transition, the scaling is criterion- independent, showing that our trial function describes the H_{c2} dependency. Deviations below midpoint are due to correlated pinning and a differing H_{irr} dependency.
